# Validation of a novel virtual reality simulation system with the focus on training for surgical dissection during laparoscopic sigmoid colectomy

**DOI:** 10.1186/s12893-021-01441-7

**Published:** 2022-01-08

**Authors:** Takashi Mori, Koji Ikeda, Nobuyoshi Takeshita, Koichi Teramura, Masaaki Ito

**Affiliations:** 1 Department of Colorectal Surgery, National Cancer Center Hospital East, Kashiwa, Chiba Japan; 2 6-5-1 Kashiwanoha, Kashiwa, Chiba 277-8577 Japan

**Keywords:** Virtual reality simulator, Postoperative pain, Surgical training, Laparoscopic colorectal surgery, Internship and residency, Haptic feedback

## Abstract

**Background:**

Mastery of technical skills is one of the fundamental goals of surgical training for novices. Meanwhile, performing laparoscopic procedures requires exceptional surgical skills compared to open surgery. However, it is often difficult for trainees to learn through observation and practice only. Virtual reality (VR)-based surgical simulation is expanding and rapidly advancing. A major obstacle for laparoscopic trainees is the difficulty of well-performed dissection. Therefore, we developed a new VR simulation system, Lap-PASS LP-100, which focuses on training to create proper tension on the tissue in laparoscopic sigmoid colectomy dissection. This study aimed to validate this new VR simulation system.

**Methods:**

A total of 50 participants were asked to perform medial dissection of the meso-sigmoid colon on the VR simulator. Forty-four surgeons and six non-medical professionals working in the National Cancer Center Hospital East, Japan, were enrolled in this study. The surgeons were: laparoscopic surgery experts with > 100 laparoscopic surgeries (LS), 21 were novices with experience < 100 LS, and five without previous experience in LS. The participants’ surgical performance was evaluated by three blinded raters using Global Operative Assessment of Laparoscopic Skills (GOALS).

**Results:**

There were significant differences (*P*-values < 0.044) in all GOALS items between the non-medical professionals and surgeons. The experts were significantly superior to the novices in one item of GOALS: efficiency ([4(4–5) vs. 4(3–4)], with a 95% confidence interval, *p* = 0.042). However, both bimanual dexterity and total score in the experts were not statistically different but tended to be higher than in the novices.

**Conclusions:**

Our study demonstrated a full validation of our new system. This could detect the surgeons' ability to perform surgical dissection and suggest that this VR simulator could be an effective training tool. This surgical VR simulator might have tremendous potential to enhance training for surgeons.

## Background

Mastery of technical skills is one of the fundamental goals of surgical training for novices. In reality, explicit knowledge does not translate into an ability to perform a successful surgery. For surgical residents, the need for tacit knowledge is extremely high. Tacit knowledge is how to move the intestine and dissect the tissue, and what it looks like and should not look like after surgical dissection. Although this knowledge is difficult to teach and obtain, its transfer is an essential element in surgical training [1].

Laparoscopic approaches are now considered to be the gold standard for advanced abdominal surgery. Laparoscopic surgery (LS) has many advantages, including less postoperative pain, better cosmetic results, and a short hospital stay [2]. However, performing laparoscopic procedures requires high and special surgical skills compared to open surgery, and it is difficult for trainees to learn through observation and practice only [3, 4]. Therefore, surgical trainers have been urged to look for alternative methods to teach medical knowledge and provide procedural experience [5]. Many studies have demonstrated that trainees who practice laparoscopic skills in a simulated environment show improved mastery of those skills when tested in that same environment [6, 7].

Simulation has been a primary support for aviators ever since the first Link flight simulator. As simulators progress, pilots now experience realistic flight, making perfect takeoffs and landings. Satava first suggested a virtual reality (VR) simulator train skills in general surgery a few decades ago [8].

VR-based surgical simulation is expanding and rapidly advancing. So far, it has been developed, including cadaveric animal models or porcine, box trainers with synthetic models, and VR simulators [9]. Previous studies have established a clear benefit of VR training that transfers skills to surgeons that are measured in operating rooms [10–13].

A major obstacle for trainees to learn LS is the difficulty of well-performed dissection. Therefore, there is an urgent need for a standard method to adequately train and assess surgical residents on how to create appropriate traction on the tissue. Yamaguchi et al. suggested that the use of retracting hand and non-dominant hand play a major role in laparoscopic performance and correlate with expertise [14]. Thus, correct surgical planes of dissection can be found, leading to safe exposure of landmarks and vital structures by a systemic dissection that comprises a precise sequence of operative steps [15]. Furthermore, an inadequate dissection may leave residual nodes and lead to vascular or ureteral injury, such as the inferior mesenteric artery during laparoscopic sigmoid colectomy [16].

However, few studies have been conducted on training and evaluation of surgical dissection [17]. Although there are some VR simulators with haptic feedback that helps in training, there are none that simulate the specific way of surgical dissection in LS. Here is the first VR simulation system with haptic feedback that tried to reproduce the principles of appropriate traction on the tissue needed for surgical dissection. This requires significantly left-handed movements as would be required in reality.

We invented a new VR simulation system that integrated haptic feedback. Moreover, it can recognize tension on the tissue which an operator creates. If they cannot create proper tension on the tissue, it is not dissected in the VR simulator. This study aimed to validate this new system that focuses on improving surgical skills of dissection in laparoscopic sigmoid colectomy. Besides, it emphasizes only one scene of procedures in laparoscopic sigmoid colectomy. The scene was medial meso-sigmoid dissection, which needs highly surgical skills to create appropriate traction.

## Methods

### The participants

Fifty subjects were enrolled in the study. Out of 50 participants: 44 were surgeons working in the Gastrointestinal Surgery, Urology, and Thoracic Surgery Department, and 6 were non-medical professionals in the National Cancer Center Hospital East, Japan. Among the enrolled surgeons who usually perform LS: 18 were LSl experts who had experience with more than 100 LS, 21 were novices with experience of fewer than 100 LS, and five surgeons had never experienced LS. None of the participants has had any prior experience with the VR simulator (Table 1).

**Table 1:** The characteristics of the participants

Sex Male Female	n = 50 44 (88%) 6 (12%)
Occupation Surgeon Non-medical professional	n = 50 44 (88%) 6 (12%)
Clinical departments Gastrointestinal surgery Urology Thoracic surgery Breast surgery Head and neck surgery Plastic and reconstructive surgery	n = 44 28 (56%) 2 (4%) 5 (10%) 3 (6%) 4 (8%) 2 (4%)
The experience of laparoscopic surgery 0 1–99 ≧ 100	n = 50 11 (22%) 21 (16%) 18 (36%)

Participation was voluntary, and participants were allowed to leave the study at any time. All participants received information about the study and provided written informed consent.

### Study design

All participants were asked to perform a certain step of the surgical procedure in VR laparoscopic sigmoid colectomy. In this step, they dissected the meso-sigmoid colon for its mobilization. Three raters assessed the participants' performance. These raters were LS experts having experience in more than 100 laparoscopic surgeries. In addition, they were qualified surgeons according to the Endoscopic Surgical Skill Qualification System in Japan, which was developed in 2004 by the Japanese Society of Endoscopic Surgery [17].

The surgical procedures blindly performed in the VR simulator were evaluated using the Global Operative Assessment of Laparoscopic Skills (GOALS). GOALS score was firstly described and validated by Vassiliou et al. It includes five evaluation items: (i) depth perception, (ii) bimanual dexterity, (iii) efficiency, (iv) tissue handling, and (v) autonomy. In this study, we excluded autonomy because it was one scene of colectomy and indicated a green line for guidance in the dissection. Each item counted for 5 points: the total score was 20 points [18].

Before evaluating the participants’ performance, we tried to facilitate an interrater agreement between the three raters. They viewed and evaluated some performances in the VR simulation simultaneously. They discussed the measurements, and then a certain consensus on the GOALS score was reached. The three raters evaluated the recordings of surgical procedures using GOALS score. The median of scores rated by the three raters was used as the score of each participant.

All 50 participants were naïve to the VR simulator to avoid bias. They were given 5 min each to familiarize themselves with the instruments and simulator before performing the procedure.

### Simulation tool

**Fig. 1:**
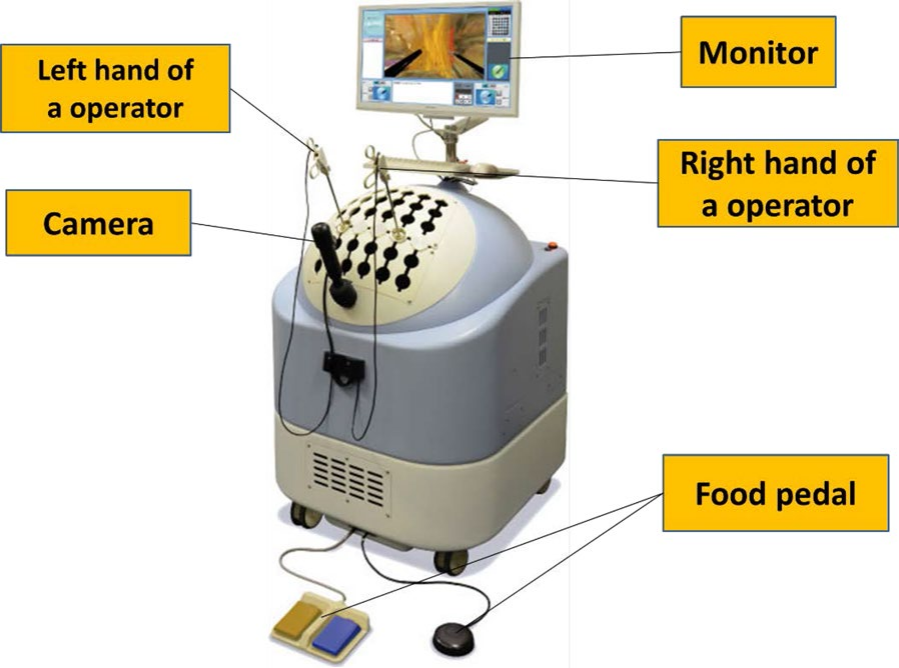
Appearance of Lap-PASS LP-100. The simulator comprises a camera and two instrumentation channels linked to a laptop computer and a foot pedal. The peritoneal cavity is virtually replicated by the software

**Fig. 2:**
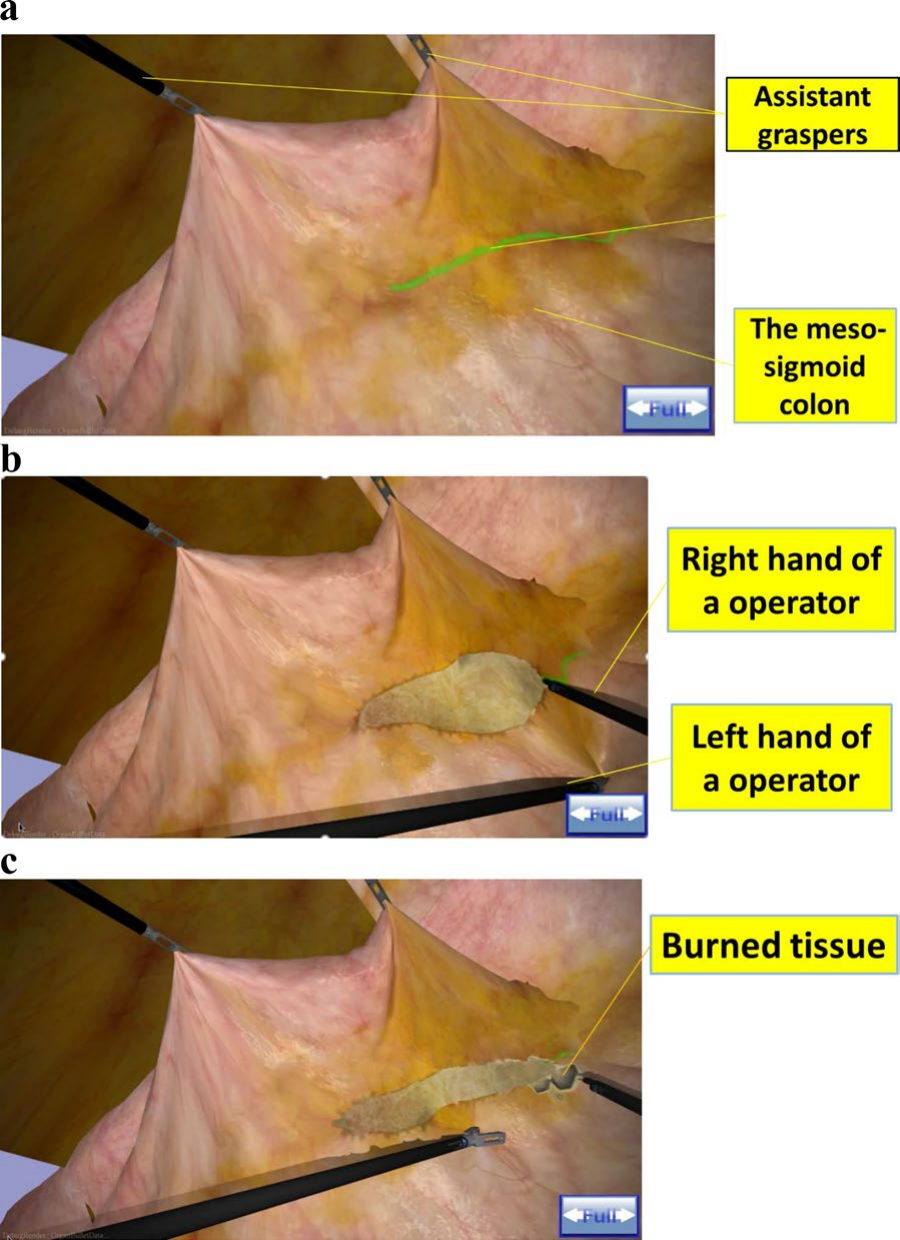
**a** The VR simulation re-indicates one scene of the laparoscopic sigmoid colectomy by a medial approach. Asterisk indicates a green line is the planned cutting line. **b** This VR simulation makes it possible to replicate the membrane structure. Regarding the cutting procedure, you can successfully cut the membrane only when the left hand creates sufficient tension in the tissue. **c** A clear cut cannot be achieved and burning will occur with insufficient tension. Burned tissue turns black. When you create too strong tension, the tissue will tear. You get force feedback if you create tension on the tissue

We developed a new VR simulation system, Lap-PASS LP-100 (Mitsubishi Precision Co., Ltd) (Fig. 1). This simulator includes training for: hand–eye coordination, depth perception, tactile perception of organs, tactics for expansion, and retention of the operative field. This was first introduced as a patient-specific simulator for LS. Makiyama et al. suggested that the system correctly reproduces anatomical structures and is a useful preoperative training tool [19]. The system uses actual computerized tomography or magnetic resonance imaging data to generate patient-specific models, allowing users to engage in surgical training in each patient. Lap-PASS LP-100 simulator comprises a camera and two simulation instrumentation channels linked to a laptop computer and a foot pedal. The software virtually replicates the peritoneal cavity. Surgical instruments are hybrids with real handles (Fig. 2a). The camera is manipulated by the user and can zoom and freeze, leaving both hands available for instrument use. This version of the VR simulator provides tactile feedback for surgical instruments interacting with organs. The software focuses on one scene of laparoscopic sigmoid colectomy procedure. This includes similar human plane structures to do laparoscopic training for dissection and exposure possible.

This VR simulator reproduces sensible tissues of the human body. It is known that surgeons have to create proper tension on tissues to dissect them (Fig. 2b). In laparoscopic procedures, it becomes more critical than in open surgery because the way of creating tension is limited to using a left-hand instrument. To reflect this difficulty, the new system demands that operators make traction on the tissue properly and utilize both hands in a complementary manner for optimal handling. It means that if an operator makes traction without care, tissues can tear.

Conversely, if too weak traction is created, tissues cannot be dissected and get burned, turning in black on the VR simulator (Fig. 2c). Compared to other simulators, haptic feedback in our system is a brand-new characteristic. This system is built by the simulation method for the deformation of membrane structure. Therefore, the VR simulator focuses on training to create proper tension on the tissue. Furthermore, two instruments for the assistant can also be manipulated.

### Operative procedures

Medial dissection of the meso-sigmoid colon is one of the most common procedures in advanced LS. It requires adequate dissection with appropriate tissue traction using a 2-handed technique. All participants performed medial dissection of the meso-sigmoid colon in the VR simulation. Assistant graspers lift a part of the meso-sigmoid colon for dissection in advance. They could dissect the meso-sigmoid colon along with the green line of guidance when the participants could grasp the tissue and create proper tension by a left hand (Fig. 2a). They could finish the procedure after dissecting a certain length. A trained technician who was present was asked to move the assistant's instruments by the operator. The technician could also provide guidance for simulator-related issues to reduce simulator-related variance.

### Data analysis

Data were prospectively collected and recorded. All scores were analyzed. Wilcoxon rank sum test was used to calculate the *p* values. *P* values smaller than 0.05 were considered statistically significant. All statistical analyses were performed with EZR (Saitama Medical Center, Jichi Medical University, Saitama, Japan), a graphical user interface for R 2.13.0 (R Foundation for Statistical Computing, Vienna, Austria). More precisely, EZR is a modified version of R commander (version 1.6-3) designed to add statistical functions and is frequently used in biostatistics. Results were expressed as median (InterQuartile Range).

## Results

**Fig. 3:**
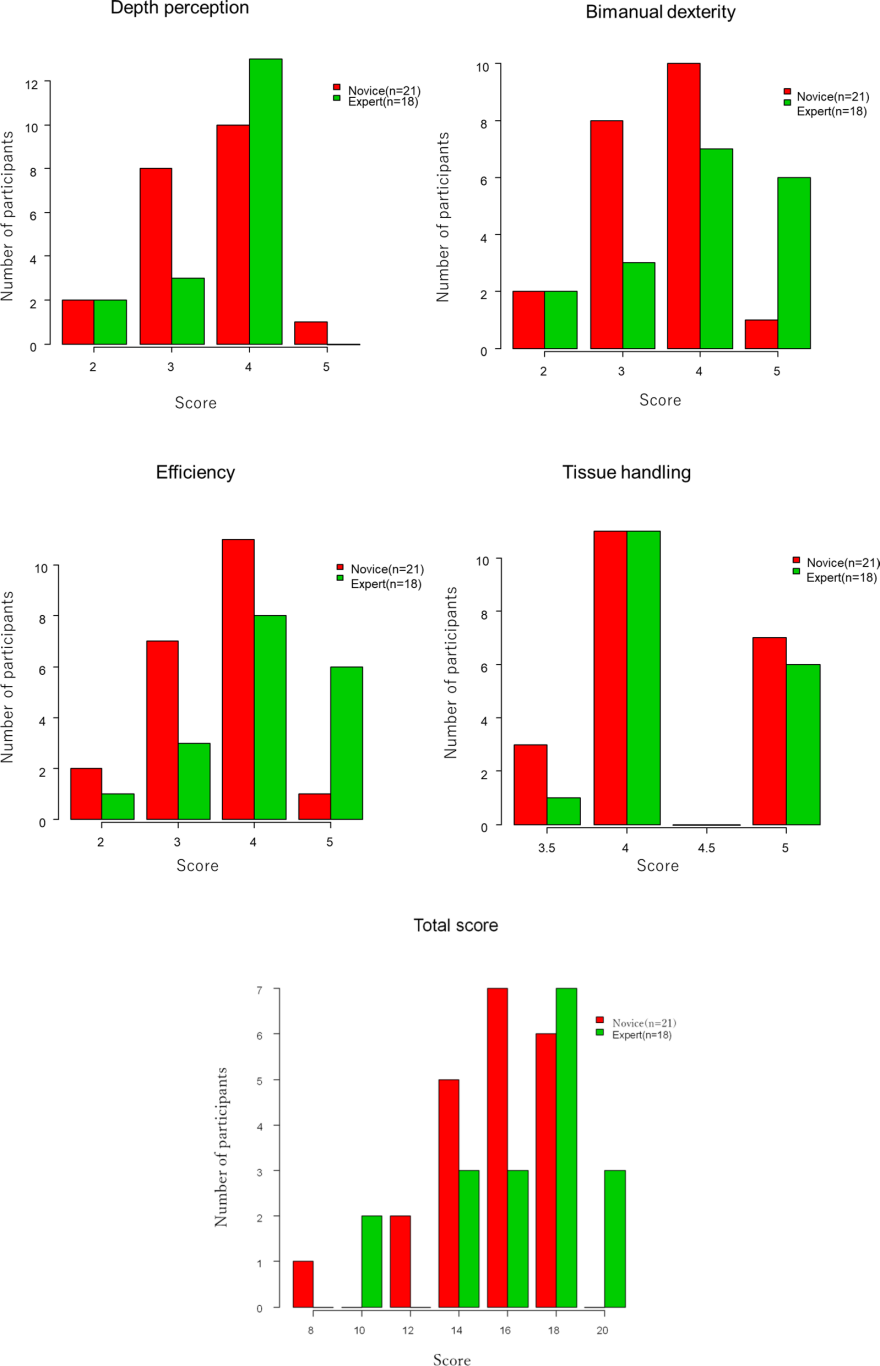
Score of each item in the novices and experts is shown by histograms

All fifty participants completed the procedure. There were significant differences in all GOALS items between the non-medical professionals and surgeons (Table 2). The total score in surgeons was significantly superior to the non-medical professionals (16.0 (14.0–17) vs. 11.5 (9.5–12), with a 95% confidence interval (CI), *p* = 0.001). Each of the 4 GOALS items also demonstrated statistically significant differences between the two groups (P values: 0.0007–0.044). The novices were surgeons who had experienced fewer than 100 LS procedures. The experts were significantly superior to the novices in one item of GOALS, efficiency ([4 (4–5) vs. 4 (3–4)], with a 95% CI, *p* = 0.042) (Table 3). There were no statistically significant differences in the other three items and total score between the novices and experts. The score of each item in novices and experts was shown by histograms (Fig. 3).

**Table 2:** Comparison of GOALS scores between the two groups: the non-medical professionals versus surgeons

	Non-medical professional n = 6	Surgeons n = 44	P value
Depth perception	2.5 (2–3)	4.0 (3–4)	0.0036
Bimanual dexterity	2 (1.25–2)	4 (3.0–4)	0.00065
Efficiency	2.5 (2–3)	4 (3–4)	0.0018
Tissue handling	4 (3.25–4)	4 (4.0–5)	0.044
Total score	11.5 (9.5–12)	16.0 (14.0–17)	0.001

Median (IQR) Wilcoxon rank sum test

Values are medians, with interquartile range in parentheses

**Table 3:** Comparison of GOALS scores between the two groups: The novices versus experts

	Novices (n = 21)	Experts (n = 18)	P value
Depth perception	4 (3.0–4)	4 (3.25–4)	0.39
Bimanual dexterity	4 (3.0–4)	4 (3.25–5)	0.080
Efficiency	4 (3–4)	4 (4–5)	0.042
Tissue handling	4 (4–5)	4 (4–5)	0.73
Total score	15 (14.0–17)	17 (14.25–18)	0.070

Median (IQR) Wilcoxon rank sum test

Values are medians, with interquartile range in parentheses

## Discussion

This study demonstrated that the new VR simulation system could significantly show differences between the surgeons and non-medical professionals. No statistically significant difference could be demonstrated for GOALS score between the novices and experts, except for one item. However, both bimanual dexterity and total score in the experts were not statistically different, although they tended to be higher than those in the novices. This result revealed that the novel simulator could detect the ability of an operator for surgical dissection.

Some laparoscopic surgical simulators were validated in previous studies, such as the *LapSim and LAP Mentor* [20–22]. They were more valuable as a training tool than an inanimate box trainer [23]. Multiple studies have been conducted to validate VR simulators as tools to train surgeons for laparoscopic skills [9]. Some VR simulators provide haptic feedback and verify the validity of these systems. Haptic, or “force-feedback” technology in VR simulation, is a rapidly developing field. Haptic feedback improves the fidelity, realism, and training effect of VR simulators. Six randomized controlled trials demonstrated that haptic-enhanced VR simulation is significantly more effective than those without haptics for skill training, particularly in novices [24]. Haptic feedback in actual operation is one element of the essential tacit knowledge that surgeons must gain. This knowledge leads to effective and accurate surgical dissection, minimal injury to adjacent organs, and less unnecessary coagulation. To the best of our knowledge, there is no other VR simulator with haptic feedback that attempted to train principles of appropriate traction on the tissue that is needed for surgical dissection.

Few studies have been conducted on training and evaluation of surgical dissection. Matsuda et al. conducted a study on it and, they concluded that motion analysis of surgical performance, such as dissection, is a powerful tool for basic skill assessment [17]. By measuring instrument tip force, Yoshida et al. found that applying a vertical force first, followed by a horizontal force with minimal vertical force, was an effective and safe method for surgical dissection [25]. However, these surgically haptic cues have not been reproduced and practiced in any training simulator.

It is challenging to evaluate surgical skills objectively. Previous studies reported validated simulator metrics, including time taken, the total number of movements, and total path length, which were objectively evaluated by specific systems in a simulator [7, 23, 26, 27]. They are useful and easy to assess by simulators. However, these metrics are summative to assist trainees in developing a concrete understating of their technical skills. Alternatively, there are global rating scales for intraoperative technical skills such as GOALS, objective structured assessment of technical skill, and operative performance rating scale. In this study, GOALS was used in evaluation, which was developed to fulfill the need for objectively quantifying surgical skills in LS. Hogle et al. demonstrated that GOALS was able to differentiate novice fellows from graduating fellows [28].

The VR simulator was designed to focus on one procedural scene in laparoscopic sigmoid colectomy. The scene is the medial meso-sigmoid dissection. Owing to the oncologic safety concerns of laparoscopic colorectal surgery (LCS), the dissemination of this technique has been slow [29]. However, surgical access remains poor for residents in LCS, as primary operators [30]. Several studies have assessed the simulation for basic laparoscopic skills and procedures. They suggested a remarkable lack of available data on the educational value of simulated training in advanced LS, such as LCS, exists [31]. The VR simulator was directed to surgical dissection, which is one of the most basic surgical skills. This could be practical training for LCS.

Today, the coronavirus disease 2019 pandemic has severely impacted healthcare systems worldwide. In addition, it has forced surgical residents to expose fewer surgical cases by redeploying intensive care and emergency and reducing elective surgical cases [32]. With a stunning reduction in operative exposure, it is challenging for surgical trainees to improve their surgical skills and knowledge. Owing to this, surgical simulators, such as the VR simulator, have the enormous potential to increase the opportunity for training instead of an actual operating theater.

Differences between the novices and experts were not statistically significant except for one item, which was efficiency. This result could reflect less sensitivity in “tissue handling” in the present simulator. The non-medical professionals received a high score of 4 (3.25–4) in tissue handling, although their scores for other items were lower than this. In a previous study, which was evaluated by GOALS, tissue handling and depth perception were not statistically significant. The learning curve in tissue handling reached a plateau at a low level in the literature [28]. It may imply the difficulty in assessing a skill of tissue handling according to GOALS. This can also explain that the study could not prove the significant difference in the total score between the novices and experts. Their scores are very near in each item.

This study had several limitations. First, the study was performed in a single center. Although this was enough to indicate a statistical significance between the non-medical professionals and surgeons, a large sample size is recommended. Second, although the assessment was performed by three laparoscopic experts after discussing and reaching a certain consensus on the evaluation of GOALS score, and the GOALS score was well-validated and showed good interrater reliability, the interrater reliability was not analyzed in this study. Third, the variability in gender was not accounted for and could have affected the outcomes. All of the non-medical professionals were female, whereas all of the surgeons were male. Fourth, we utilized incomplete GOALS score for evaluation. However, we excluded one out of five items, which was autonomy, because this task could not evaluate autonomy in the participants.

## Conclusion

In conclusion, our study demonstrated the validation of our new VR simulation system that simulates surgical dissection in the tissue during LS. It suggests that this simulator can be an effective training tool for surgeons. This surgical VR simulator might have the potential to enhance training for surgeons.

## Data Availability

The datasets used and/or analyzed during the current study are available from the corresponding author on reasonable request.

## Abbreviations

VR: Virtual reality
LS: Laparoscopic surgery
GOALS: Global Operative Assessment of Laparoscopic Skills
LCS: Laparoscopic colorectal surgery
